# Influence of nickel hydroxide catalyst ink formulation on direct bar coating of anion exchange membranes†

**DOI:** 10.1039/d4ra03969f

**Published:** 2024-12-10

**Authors:** Mohamed Elshamy, Lukas Metzler, Joey Disch, Severin Vierrath, Susanne Koch

**Affiliations:** a Electrochemical Energy Systems, IMTEK Department of Microsystems Engineering, University of Freiburg Georges-Koehler-Allee 103 79110 Freiburg Germany susanne.koch@imtek.uni-freiburg.de; b Hahn-Schickard Georges-Koehler-Allee 103 79110 Freiburg Germany

## Abstract

Achieving uniform and high-performing catalyst-coated membranes (CCMs) is a critical challenge in the field of electrochemical energy conversion technologies. This challenge is particularly pronounced in the coating of catalyst inks, where optimizing ink formulations and mixing conditions is essential for producing homogeneous catalyst layers that enhance electrochemical performance. In this study, we investigate the influence of mixing parameters and solvent composition on the rheological behavior and performance of nickel hydroxide-based anode inks, specifically for application in anion exchange membrane (AEM) electrolysis. We systematically explored the effects of roller mixing speed (30 and 80 rpm), mixing duration (4, 24, and 48 hours), and water content (0, 25, 34, and 51 %) on the morphology and homogeneity of the catalyst layers. Our findings reveal that a mixing speed of 80 rpm and a duration of at least 24 hours are necessary to achieve desirable catalyst layer morphology. Additionally, the presence of water in the ink formulation was critical, with an optimal water content of 34 % (3 : 1, water : IPA) yielding the best morphological homogeneity and reproducible electrochemical performance. The study demonstrates a 70 mV reduction in overpotential, resulting in a voltage of 2.05 V at a current density of 1 A cm^−2^. While mixing parameters minimally impacted the rheological behavior of the inks, they significantly influenced the electrochemical performance and morphology of the CCMs.

## Abstract 2

To achieve uniform and high-performing catalyst-coated membranes for anion-exchange membrane water electrolyzers using Ni(OH)_2_ careful ink optimization and analysis is necessary.

## Graphical abstract

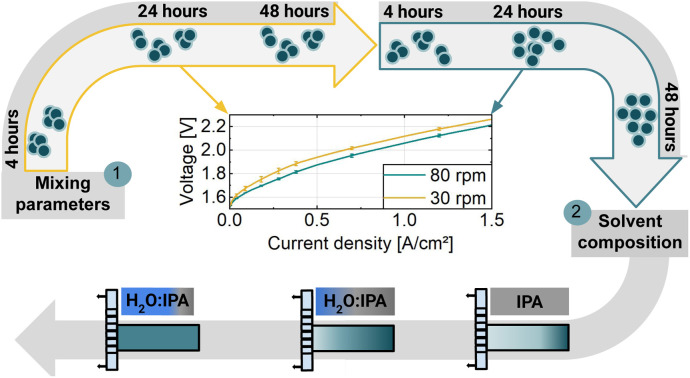

## 1. Introduction

Amid the intensely growing interest in green hydrogen production, anion exchange membrane (AEM) water electrolysis has garnered significant attention due to its unique capability of combining the main advantages of two commercial water electrolysis technologies. AEM electrolysis allows the use of low-cost, abundant catalysts, as seen in alkaline water electrolysis, while also achieving high current densities similar to proton exchange membrane water electrolysis.^1,2^ Nickel-based oxygen evolution reaction (OER) catalysts exemplify these low-cost materials, having demonstrated superior electrochemical performance compared to the scarce and expensive iridium oxides in alkaline media.^3–5^ Despite this progress, identifying an effective substitute for carbon-supported platinum black catalysts for the hydrogen evolution reaction (HER) remains an ongoing challenge.^6^

Ink mixing methods are usually based on ball milling, for example roller and planetary mixers.^7,8^ Roller mixers are simpler, cheaper and require less maintenance, however, due to their relatively low speed, they require several hours up to two days for ink mixing.^7,9^ While parameters like solvent composition, mixing time and speed can have a strong effect on ink homogeneity and thus cell performance, the exact influence remains unclear.

Coating these catalysts directly onto a membrane *via* bar coating has emerged as a promising approach, yielding catalyst layers with state-of-the-art performance that can be scaled for production.^9,10^ Unlike layer-by-layer spray coating, direct one-step deposition on the membrane demands inks with moderate to high viscosity, depending on the coating technique.^7,9^ Bar coating, a type of Mayer rod coating, is a popular method due to its ability to generate large-area films efficiently and cost-effectively. The wet thickness of the coating is proportional to the wire diameter of the Mayer rod, and surface tension and viscosity of the ink play critical roles in achieving uniform coatings. Factors such as wetting, leveling, and dewetting are strongly influenced by these properties.^11^

The microstructure of the catalyst layer (CL) formed during coating is crucial for performance, as it impacts electrode kinetics, mass transport, and overall cell efficiency. The CL microstructure, governed by the interactions between catalyst particles and ionomer within the ink, determines key characteristics such as catalyst-ionomer interaction, ionomer phase continuity, and pore size distribution.^12^ The microstructure is significantly influenced by the ink's rheological properties, which in turn depend on factors like solvent composition, mixing time, and mixing speed. Rheology serves as a sensitive technique to characterize the bulk microstructure of inks, providing insights into how these properties affect coating uniformity, thickness, and ink penetration into porous substrates, as well as the final CL morphology after drying.^13^ Thus, understanding and optimizing the rheological behavior of catalyst inks is critical for establishing effective materials-processing-performance relationships in electrode fabrication.

In this study, we focus on optimizing the ink fabrication process for nickel hydroxide (Ni(OH)_2_)-based catalysts as part of the catalyst-coated membrane (CCM) architecture. Ni(OH)_2_ is commercially available, cost-effective, and demonstrates acceptable electrochemical performance, making it an ideal candidate for research purposes. The study was conducted in two phases: the first phase varied roller mixer parameters while maintaining a consistent ink composition with deionized water (DI-H_2_O) as the primary solvent. The second phase explored the effect of solvent composition by adjusting the DI-H_2_O to isopropanol (IPA) ratio. We investigated the rheological behavior of the inks, followed by *ex situ* and *in situ* characterization of the resulting catalyst layers.

## 2. Methods

### 2.1. Materials

Anion-exchange ionomers (AP3-HNN9-00-X, Lot: JMY220604) and anion-exchange membranes (AF3-HNN9-50-X, reinforced, 50 μm, Lot: A30425C01 & Lot: 2B16R01) were provided by Ionomr Innovations Inc. Ni(OH)_2_ powder (Merck, SKU: 283622) and Pt/C (50 wt %, Elyst Pt50 0550) were used as anode and cathode catalysts, respectively. Ethanol (EtOH, 99.5 % Ph. Ezr., extra pure), (IPA, 70 %, pure) were purchased from Carl Roth GmbH + Co. KG. Potassium hydroxide (KOH) pellets (85 %, VWR) were used to prepare 1 M KOH electrolytes. Nickel fiber felts (BEKIPOR® 2NI06-0.20), were purchased from Bekaert and Freudenberg H24C5 carbon paper with a microporous layer was purchased from FuelCellStore. Polytetrafluoroethylene (PTFE) sheets were purchased from Böhme-Kunststofftechnik GmbH & Co. KG as gaskets and spacers.

#### 2.1.1. MEA fabrication

Cathode inks for spray coating contained Pt/C 50 wt % (180 mg) dispersed in 4.838 g DI-water and 3.69 g IPA. After adding the previously prepared ionomer solution (0.9 g, 5 wt % ionomer and 0.9 g EtOH/Acetone 1/1), the ink was sonicated at an amplitude of 90 % with 0.7 cycle frequency for 40 minutes.

Spray coated gas diffusion electrodes (GDE) were coated with the Pt/C ink using an ultrasonic spray coater (SNR 300, Sonocell) adapting a procedure published previously.^1,14^ The GDEs were held at 40 °C and catalyst layers were deposited slowly (0.3 ml min^−1^ flow rate with a nozzle speed of 140 mm s^−1^) and with a 5 s pause between each layer to allow the layer to dry. Spray coating was done until a loading of 0.4 mg_pt_ cm^−2^ was achieved as measured by weighing a control substrate that was placed next to the GDEs.^14^

Anode inks for bar coating contained Ni(OH)_2_ dispersed in DI-water (0.9 g). After addition of the previously prepared ionomer solution (0.45 g, 5 wt % Aemion+-HNN9 in EtOH/acetone 1/1, w/w) and (0.45 g of a EtOH/acetone solvent mixture 1/1, w/w), ZrO_2_ grinding balls (Retsch 22.455.0009) were added. This resulted in an overall solid content of 15.36 %. The inks were then placed on a roll mixer for various time intervals (4, 24 and 48 hours) at two set rolling speeds (30 rpm and 80 rpm). The Ni(OH)_2_ inks were then deposited on the membranes *via* bar coating, producing so called half catalyst coated membranes (HCCM).

Bar coated HCCMs were prepared as previously described by Koch & Metzler *et al.*, adapting the procedure to coating only one side of the membrane.^9^ A bar coater (Thierry, PG-032-320150) with nominal wet film thickness of 150 μm and a casting table (Zehntner by proceq, ZAA 2300) were used to coat the membrane at a speed of 40 mm s^−1^. The HCCM was subsequently left to dry in the fume hood at room temperature for at least 24 hours. Each HCCM was fabricated with a freshly made catalyst ink and three samples were cut from each HCCM to measure identical samples for the determination of error bars.

For the solvent variation study, the same ink with different water contents have been synthesized. In addition to the ethanol and acetone used in the ionomer solution, DI-H_2_O to IPA ratios were varied to synthesize four different inks. Starting with only DI-H_2_O (100 % H_2_O) as per the standard ink, DI-H_2_O : IPA 1 : 1 (50 % H_2_O), DI-H_2_O : IPA 3 : 1 (75 % H_2_O) and only IPA (0 % H_2_O). The ionomer to catalyst ratio was fixed to 0.08 and the solid content to 15.36 %. All inks were mixed on the roller mixer at 80 rpm for 24 hours.

#### 2.1.2. Electrochemical measurements

Each HCCM and a GDE (5 cm^2^ active area) were exchanged into the hydroxide form by first, immersing in 1 M NaCl/1 M KOH, 9/1 w/w for 24 hours and then in 3 M KOH for 24 hours followed by 1 M KOH for 1 hour before assembly, to neutralize the KOH concentration to that of the utilized liquid electrolyte. The electrolyte was prepared by adding 99.91 g of KOH to 1.5 liters of DI-water, resulting in a 1 M KOH electrolyte solution, which is then used for the anolyte and catholyte.

For electrochemical measurements, three cells were measured for each variation, except for the cell with 50 % DI-H_2_O, where one cell encountered an operational failure. The overall trend remained, however, unaffected. The prepared electrodes were assembled in a MEA sandwiched between two flow fields, connected to two electrolyte tanks and to a potentiostat (BioLogic VSP300 with two 10 A/5 V amplifiers). The electrochemical characterization protocol started with preconditioning steps followed by galvanostatic electrochemical impedance spectroscopy (GEIS) as follows: (1) 10 min step of electrolyte heating up and circulation, (2) voltage ramp up to 1.8 V then down to 0 V, (3) 1 min resting period, (4) two consecutive GEIS measurements of 0.5 and 1 A, at frequencies from 100 kHz to 100 mHz, (5) a polarization curve was measured *via* current density step values according to the EU harmonized measurement protocol as previously published by Koch *et al.*^1^ (6) same as step 4 but with applied currents of 2.5 and 5 A, (7) second polarization curve as step 5. Unless otherwise noted, the first polarization curve (#5) is displayed as electrochemical performance. The safety limit was set to 2.3 V for all measurements, which means that the highest achieved current density was at a voltage of 2.3 V.

#### 2.1.3. X-ray fluorescence microscopy

A Bruker M4 Tornado X-ray fluorescence microscope (μXRF) was used to determine the approximate loading of the Ni(OH)_2_ as well as Pt/C catalysts on the HCCMs and GDEs, respectively. The loading was extracted using the Bruker XMethod software, modelling the sample layers. Due to sensitivity of the instrument on the focal level, unevenness of the HCCMs can introduce a systematic inaccuracy, which is mitigated by taking the average of 3–5 points on an approximately 4 × 4 cm^2^ area of the HCCM, while refocusing for each point.

#### 2.1.4. Scanning-electron-microscopy/energy-dispersive-X-ray-spectroscopy (SEM/EDX)

Samples for SEM/EDX imaging were prepared by cutting small pieces from the CCMs and fixing them on SEM studs using double-sided adhesive carbon pads. Images of the Ni(OH)_2_ electrodes were taken at 10 kV using a scanning electron microscope (SEM, MAIA 3) and a SE detector.

#### 2.1.5. Rheological characterization of the Ni(OH)_2_ inks

Viscosity, frequency sweep and oscillation measurements of Ni(OH)_2_ inks were carried out using a rheometer (MCR 101 Anton Paar). The inks were taken off the roller mixer and used directly without settling time. A volume of 500 μl of ink was deposited on the measuring surface directly under a parallel measurement plate with a diameter of 25 mm. The measurement gap was set to 0.5 mm to make sure that the distance between the sample and the measurement plate is significantly larger than the largest particle/agglomerate inside the ink as per manufacturer's recommendation. The excess ink was then trimmed off and a solvent trap was used to minimize evaporation of the solvents during measuring. The temperature was set to 20 °C for all measurements.

For the viscosity measurements, a pre-shear step was applied at 500 s^−1^ shear rate for 30 s to eliminate any load history, then the sample was left to rest for 30 s. Subsequently, a viscosity sweep from 1000 to 0.001 s^−1^ shear rate was carried out and recorded.

For frequency sweeps, the storage and loss moduli were measured at a constant strain amplitude of 5 % across a range of angular frequencies from 1 to 600 rad s^−1^. Strain sweeps were conducted at a constant angular frequency of 10 rad s^−1^, where the storage and loss moduli were determined for varying strain amplitudes between 1 and 100 %.

## 3. Results and discussion

**Fig. 1:**
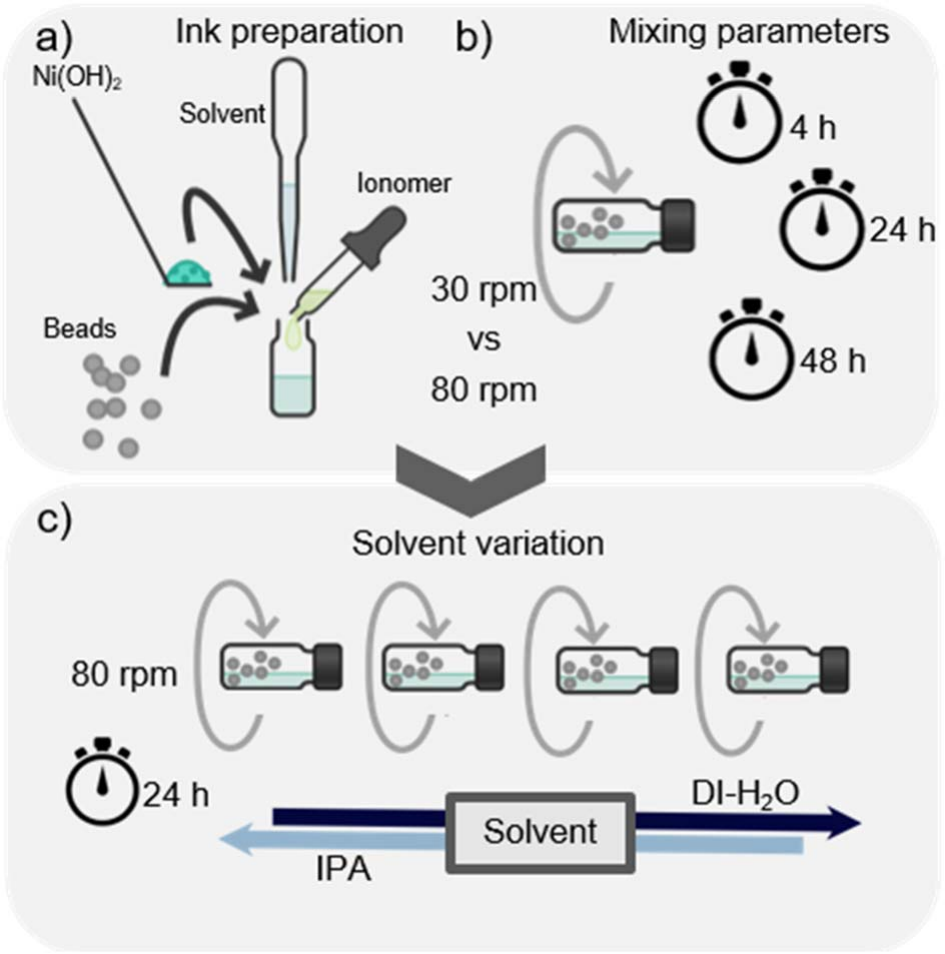
(a) Ink mixing process, (b) description of the mixing parameters variation process, and (c) description of the primary solvent variation process. Created with Chemix.

The aim of this study was to systematically investigate and improve the homogeneity and the electrochemical performance of the anode catalyst layer fabricated *via* direct bar coating. The study consisted of two phases as shown in Fig. 1. Firstly, the mixing parameters of the ink were varied and secondly, the primary solvent (DI-H_2_O) of the ink was substituted by a DI-H_2_O and IPA mixture. For both variations, rheological behavior of the inks was investigated, followed by morphological and finally electrochemical characterization.

### 3.1. Mixing parameter variation

#### 3.1.1. Effect on ink rheology

Ni(OH)_2_ inks mixed for 4, 24 and 48 hours at two different mixing rates (30 and 80 rpm) were characterized using rheometry. All inks exhibit a similar shear thinning behavior with similar viscosities under most of the applied shear rate range (see Fig. S1†). The shear thinning is attributed to the break-down and rearrangement of agglomerates upon shear application.^15,16^ However, different viscosities at lower and higher shear rates suggest the presence of agglomerates with different sizes and different hydrodynamic bonding.^15^ For instance, in the low shear rate region the inks mixed at 30 rpm for 4 hours and 48 hours have a higher viscosity and shear thinning magnitude than those mixed at 80 rpm. This could be attributed to the higher porosity and larger agglomerates of inks mixed with 30 rpm, leading to an increase in the effective volume, and hence to an increase in viscosity.^15^ This can also be seen in light and electron micrographs (Fig. S5 and S6†), where the catalyst layers from all inks have relatively large pores and larger catalyst/ionomer agglomerates, except for the ones mixed at 80 rpm for 24 and 48 hours.

Further decreasing the shear rate, the viscosity continues to increase even at very low shear rates which is known as yield stress. This suggests that the inks form a 3D percolated network in contrast to isolated agglomerates as mentioned by Khandavalli *et al.* for carbon-based inks.^15^

**Fig. 2:**
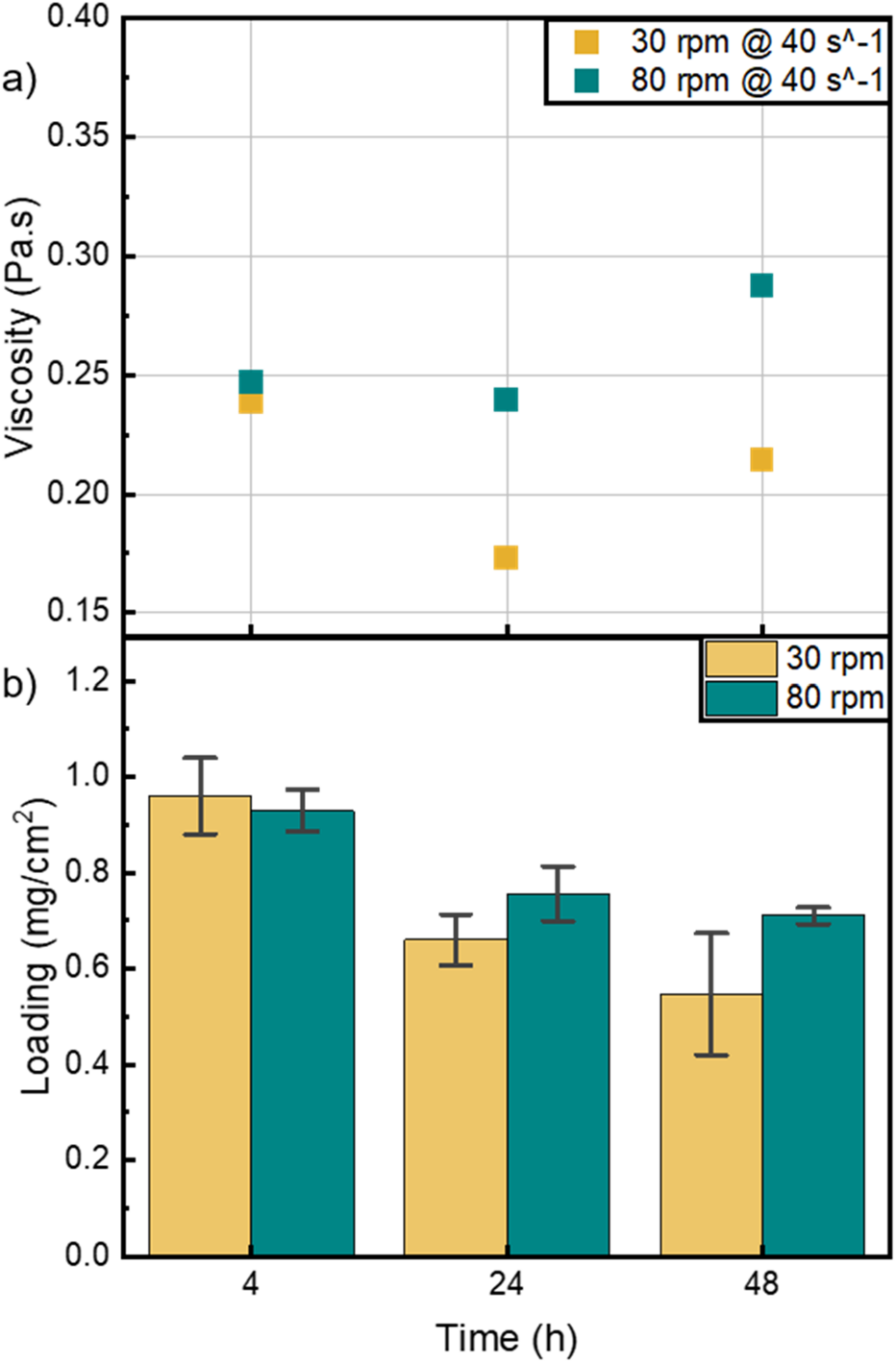
(a) Relative viscosity of inks mixed at 30 (yellow) and 80 rpm (green) and for 4, 24 and 48 hours at a shear rate of 40 s^−1^. (b) Mass loading of nickel in the catalyst layers determined by the μXRF measurement. Error bars represent the variation in the loading determined for the three cells cut from each fabricated HCCM.

The bar coating window, as mentioned by Dan *et al.*, specifies that viscosity values at shear rates between 10 and 100 s^−1^, should vary between 0.01 and 1 Pa s for optimum coating properties.^10^Fig. 2a shows the relative viscosities of the different inks at a shear rate of 40 s^−1^, where the inks showed no striking difference. This indicates that all inks have similar rheological characteristic in the shear rate regime relevant to the bar coating technique. However, the abovementioned viscosity behavior of the different inks at lower shear rates acts as a pre-indicator to the catalyst layer morphology.

#### 3.1.2. Effect on the homogeneity of the catalyst layers

To study the homogeneity and distribution of catalyst on the half catalyst coated membranes (HCCMs), the nickel loading was determined using μXRF. Fig. 2b shows that the overall nickel loading is decreasing with increasing mixing time. The width of the error bars can be seen as an indicator for the catalyst layer homogeneity. The loading variations shown by the error bars for the 30 rpm inks are likely caused by measurement inaccuracies due to sample height variations and inhomogeneity in the catalyst layer. For example, some measurement points may have been within pores in the catalyst layer, while others were on larger agglomerates. The decrease in the overall nickel loading with increasing mixing time agrees with the rheological behavior of the inks, where inks mixed for 48 hours exhibited a higher shear thinning behavior at high shear rates >100 s^−1^ (Fig. S1†).

The effects of mixing speed and mixing time can also be seen in the images of the HCCMs taken by SEM and by a microscope with 5× magnification, (Fig. S5 and S6†). Overall, the visible agglomerates are becoming smaller with longer mixing time, and the pores are getting smaller in size and fewer in number (Fig. S5a–c†). In Fig. S5d–f,† the inks mixed at 80 rpm show a significant improvement in the catalyst-pore distribution from 4 to 24 hours and less prominent from 24 to 48 hours. Furthermore, inks mixed at 30 rpm exhibited improved morphological homogeneity after 24 hours compared to those mixed for just 4 hours. However, even after 48 hours, the 30 rpm inks did not achieve the level of morphological homogeneity observed in inks mixed at 80 rpm for 24 and 48 hours.

#### 3.1.3. Effect on the electrochemical performance

**Fig. 3:**
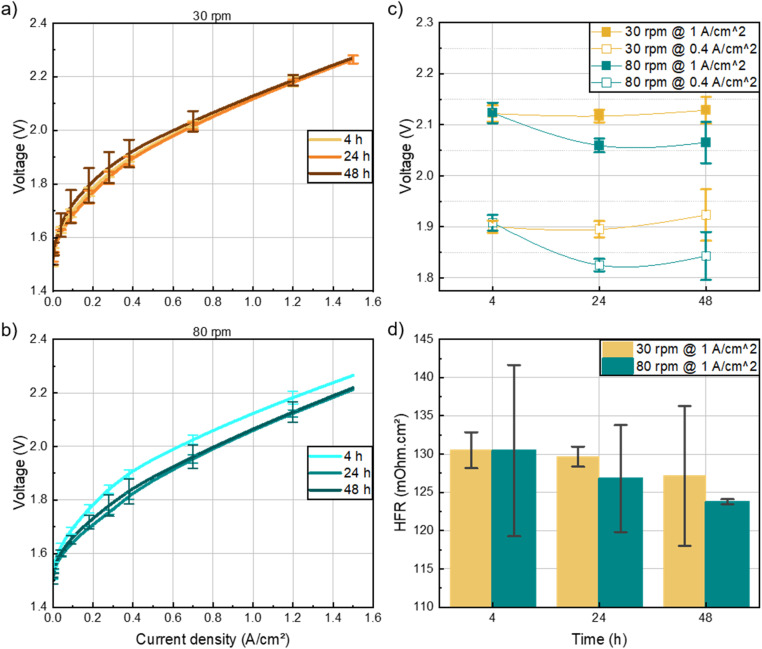
(a) Polarization curves of all cells fabricated with inks mixed at 30 rpm, at three mixing time intervals, shown in brown variations, (b) those mixed at 80 rpm, cyan variations, (c) voltage at 0.4 and 1 A cm^−2^ for 30 rpm (yellow) and 80 rpm (cyan). The error bars represent the variation between identically measured cells cut from the respective HCCM. All cells are measured at 60 °C with 1 M KOH liquid electrolyte with identical materials and methods except for the denoted anode variation.

In this section the impact of ink mixing time and speed on cell performance in a single-cell AEM water electrolyzer is discussed. Despite the noticeable morphological differences in the catalyst layers of the 30 rpm inks, the electrochemical performance did not seem to be significantly affected (Fig. 3a), particularly for the inks mixed for 4 and 48 hours. This might be due to the persistence of pores and relatively large agglomerates even after 48 hours of mixing, which could compensate for other factors affecting electrochemical performance.

At high current densities, the performance of cells for the varied mixing time converged, suggesting that the effect of mixing time at 30 rpm is negligible on the cell performance at relevant operational current densities (*i.e.* ≥1 A cm^−2^). On the other hand, Fig. 3b shows that for the 80 rpm inks, the smooth morphology of the catalyst layer obtained after 24 hours of mixing appeared to have a significant impact on the electrochemical performance of the cells. Specifically, the 24 hours ink demonstrated a 70 mV reduction in overpotential at 1 A cm^−2^ compared to the 4 hours ink (Fig. 3c). Notably, the performance plateaued between 24 and 48 hours, indicating that 24 hours of mixing is sufficient to achieve optimal results. The improvement in the electrochemical performance for the 24 and 48 hours, 80 rpm-cells can also be attributed to the high frequency resistance (Fig. 3d). The lower cell overpotential and HFR could be due to the better interconnectivity of the catalyst-ionomer agglomerates within the layer as depicted in the SEM images (Fig. S5†). As the HFR of the cells was the same at 0.4 A cm^−2^ and 1 A cm^−2^, only the HFR at 1 A cm^−2^ is shown. Based on these findings, all subsequent inks were mixed at 80 rpm for 24 hours to achieve optimal performance while minimizing fabrication time.

### 3.2. Water content variation in the ink

The homogeneity and coatability of a catalyst layer fabricated *via* bar coating is highly dependent on the ink's viscosity.^10^ A homogeneous layer is crucial for consistent performance across large batches and active areas of CCMs.^16,17^ To explore how ink rheology impacts bar-coated HCCMs, we altered the solvent composition by adjusting the ratio of deionized water (DI-H_2_O) to isopropanol (IPA) in the primary solvent, which was initially just DI-H_2_O in the first phase of the study. The secondary solvents which are ethanol and acetone from the ionomer solution, remained unchanged. All inks were mixed at 80 rpm for 24 hours as concluded from the section above.

#### 3.2.1. Effect on the rheology of the Ni(OH)_2_ inks

**Fig. 4:**
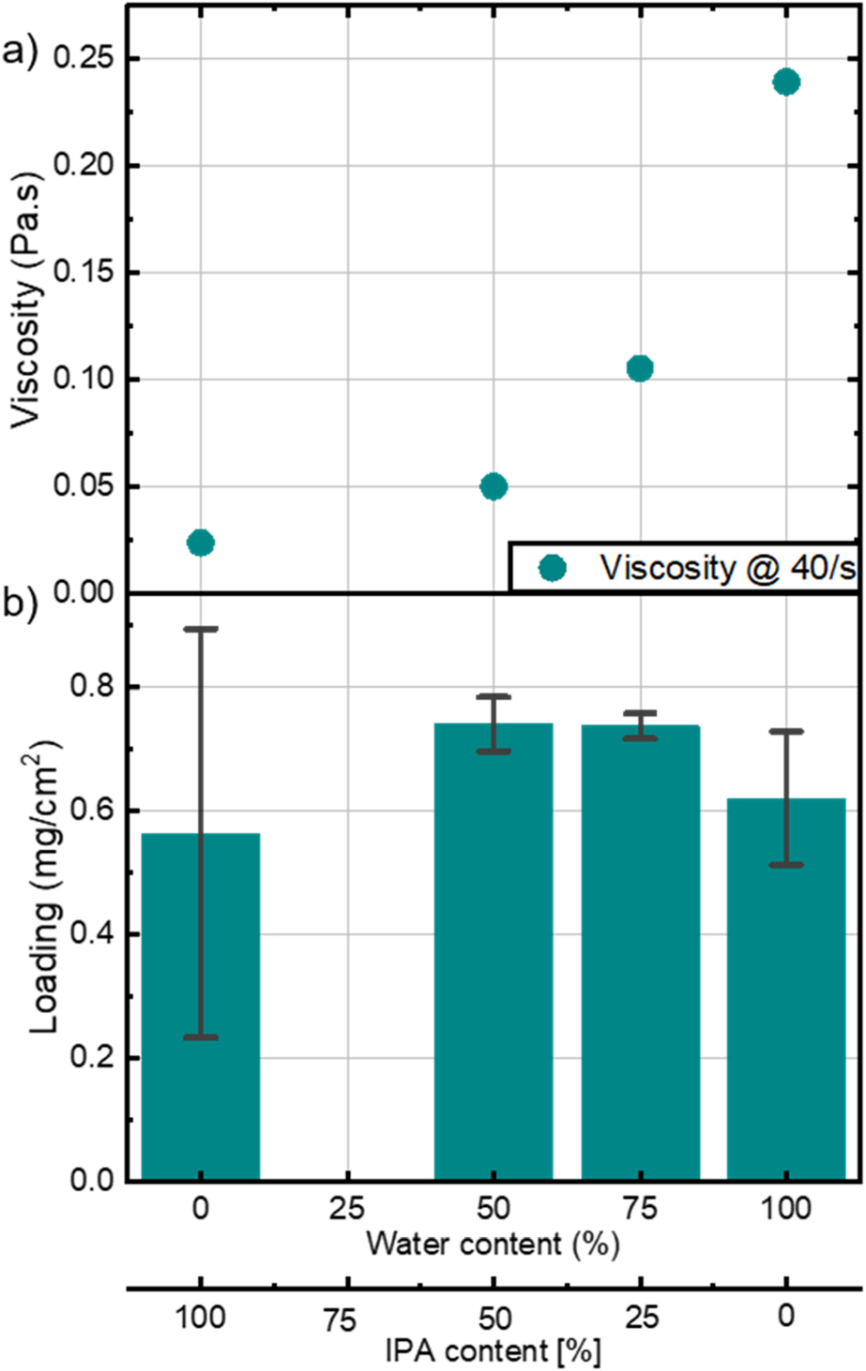
(a) Viscosity of inks with varying water content at a shear rate of 40 s^−1^. (b) Ni mass loading on HCCMs coated with inks of different water contents/IPA contents. Error bars represent the variation observed among the three extracted cells from each HCCM.

Fig. 4a shows the changes in relative viscosity of the inks with varying water to IPA ratio at a fixed shear rate of 40 s^−1^, this shear rate lies within the coating window as mentioned by Dan *et al.*^10^ A relative viscosity increase by a factor of ten can be seen in Fig. 4a between the ink with 0 % water and that of 100 % water. Given that pure water/IPA mixtures with different water contents differ only slightly in viscosity,^18^ it seems that the significant increase in viscosity accompanied with water content increase is attributed to the interaction between the ionomer and water molecules and/or water molecules and hydroxide ions from the Ni(OH)_2_ catalyst. All inks, except for the ink with 0 % water, fall within the coating window. Remarkably, the impact of water content on the ink's rheological behavior is not the same for all catalyst inks, for example, carbon-supported Pt inks used in fuel cells, show no significant change when varying ratio of water and IPA as studied by Guo *et al.*^19^ There, an increase in relative viscosity of less than 10 % is observed, when the water content is increased from 5 to 80 %.^19^

The impact of the water content is also demonstrated in Fig. S3,† where the *y*-intercept of the shear stress curves is higher for inks with more water content. This intercept is known as the yield stress, which is high when the ink behaves less like a fluid at rest and a certain force has to be applied before the ink starts to flow.^20^ This parameter is essential for the bar coating process, to prevent the ink from spreading outside the coating area during bar coating.

Fig. S4a–c† assesses the viscoelastic behavior of the inks with different water contents. Inks containing water exhibit a viscoelastic solid behavior as storage modulus (*G*′) is bigger than loss modulus (*G*′′) and both are independent of strain at low strain values.^19^ The magnitude of the storage modulus also depicts the strength of the ink which is in this case proportional to the water content in the ink. The ink with no water content is shown to be a viscoelastic liquid, and is entirely dependent on strain, depicting the absence of a gel like inner network.^21^ Inks with 0 % water, and therefore the lowest viscosity, resulted in highly inhomogeneous layers (Fig. 4b and S7†). The error bars depict the standard deviation of the three measured cells cut from a fabricated HCCM. A mixture between water and IPA is beneficial to improve homogeneity, especially with more water than IPA. This ratio allowed to coat an area of 48 cm^2^ with very homogeneous loading (Fig. S8†). These findings highlight the importance of optimizing ink viscosity through careful solvent selection, particularly for scale-up of the CCM fabrication.

#### 3.2.2. Effect on the electrochemical performance

**Fig. 5:**
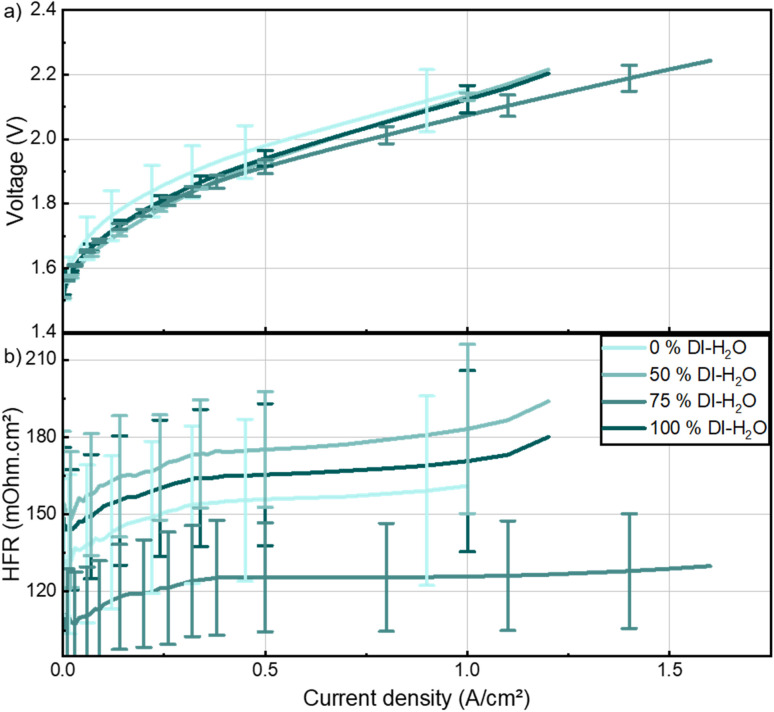
(a) Polarization curves and (b) high-frequency resistance (HFR) of cells fabricated with anode inks of different water contents. All cells are measured at 60 °C with 1 M KOH liquid electrolyte with identical materials and methods except for the denoted anode variation.

Fig. 5 shows the electrochemical performance for different water contents in the ink. The mean polarization curves are plotted (Fig. 5a), with error bars denoting the standard deviation. The maximum current density of the three cells is limited to the cell with lowest performance to be able to show error bars for the entire current density range. No significant performance trend is seen in Fig. 5. However, one cell with fabricated with a pure IPA ink showed a significantly lower performance which is potentially related to the significant variation in loading observed with this ink (Fig. 4b). Subsequently, the error bars are also an indication of the inhomogeneity of the catalyst layer. A mixture between water and IPA seems to be giving the best homogeneity and lowest spread in electrochemical performance, with the 75 % water ink having all of the three cells exceeding 1.5 A cm^−2^ under 2.3 V, with the lowest HFR values (Fig. 5b).

## 4. Conclusion

In this study, we investigated how the composition of anode inks and mixing parameters influence catalyst layer morphology and electrochemical performance in AEM water electrolysis. Through our examination of ink rheology, catalyst loading, and layer homogeneity, we found that a mixing speed of 80 rpm, combined with a mixing time of at least 24 hours, was required to achieve optimal homogeneity and electrochemical performance. Our findings emphasized that high mixing rates are essential to break up agglomerates in the catalyst inks, as increasing the mixing time alone will not suffice if the mixing rate is too low. At higher mixing rates, such as 80 rpm, the mixing time can be reduced to 24 hours or potentially further, but this needs to be individually determined for each catalyst ink.^22,23^

Additionally, our results highlighted the critical role of solvent composition, with a 75 % water content using a 3 : 1 DI-H_2_O to IPA ratio proving optimal for both homogeneity and cell performance. This underscores the importance of identifying the ideal combination of IPA and DI-H_2_O in the catalyst ink to ensure consistent results. Overall, the study highlights the necessity of thoroughly optimizing both catalyst ink composition and mixing parameters to achieve homogeneous catalyst layers and reproducible electrochemical performance. This is particularly important as we advance toward scalable fabrication of catalyst-coated membranes with large active areas, where the increased viscosity of direct bar-coating inks demands different considerations compared to those typically used for spray-coating at the lab scale. The optimized conditions may be specific to the experimental setup, particularly for nickel hydroxide-based inks and the bar coating technique in this study. Further studies are necessary to determine the universality of these findings across different materials and fabrication methods.

## Data availability

Data for this article, including raw data of electrochemical measurements is available at https://freidata.uni-freiburg.de/records/dyytp-zjv65 with DOI: 10.60493/dyytp-zjv65.

## Author contributions

Mohamed Elshamy: investigation, methodology, validation, experimental work, writing – original draft. Lukas Metzler: investigation, methodology, validation, writing – review & editing. Joey Disch: project administration, supervision, writing – review & editing. Severin Vierrath: conceptualization, writing – review & editing. Susanne Koch: conceptualization, investigation, methodology, validation, writing – review & editing.

## Conflicts of interest

The authors declare no competing financial interest.

## Supplementary Material

RA-014-D4RA03969F-s001
