# Supplementary material for: Influence of nickel hydroxide catalyst ink formulation on direct bar coating of anion exchange membranes
Source: RSC Adv. 2024 Dec 10;14(52):38996–9003. doi: 10.1039/d4ra03969f (PMC11629302; doi:10.1039/d4ra03969f)
Supplement: RA-014-D4RA03969F-s001 [file RA-014-D4RA03969F-s001.pdf]

Supplementary Information (SI) for RSC Advances.
This journal is © The Royal Society of Chemistry 2024

Supplementary information for

Influence of Nickel Hydroxide Catalyst Ink Formulation on Direct Bar Coating of Anion Exchange Membranes

Mohamed Elshamy[1], Lukas Metzler[1, 2], Joey Disch[1, 2], Severin Vierrath[1, 2], Susanne Koch[1, 2]*

1. Electrochemical Energy Systems, IMTEK Department of Microsystems Engineering, University of Freiburg, Georges-Koehler-Allee 103, 79110 Freiburg, Germany
2. Hahn-Schickard, Georges-Koehler-Allee 103, 79110 Freiburg, Germany

Rheological measurements

Six inks with different mixing parameters (time and rpm) were measured and viscosity sweeps plotted (Figure S1 and S2). Three more inks mixed at 80 rpm for 24 hours with DI-H_2O: IPA solvent variation were measured and the viscosity sweeps were plotted (Figure S2). Figure S4a-c depict the viscoelastic behaviors of the four inks with different solvent composition, and Figure S4d depicts the time-dependent behavior of the inks.

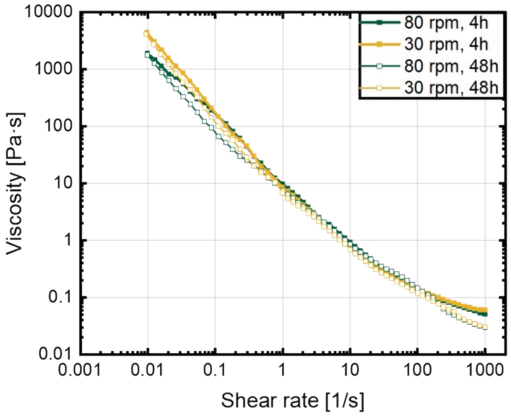

S1 Viscosity sweeps of inks mixed at 30 rpm (yellow) and 80 rpm (Cyan) for 4 and 48 hours.

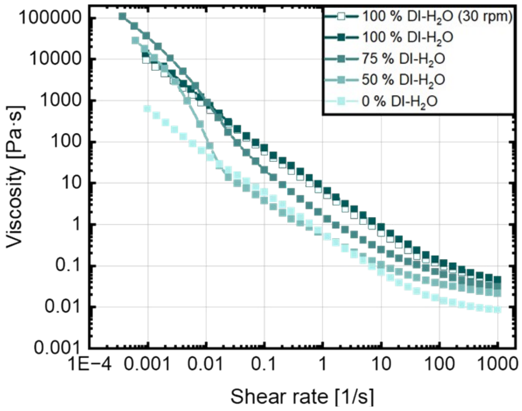

S2 Viscosity sweeps of inks with different water contents, all inks were mixed at 80 rpm for 24 hours unless otherwise specified.

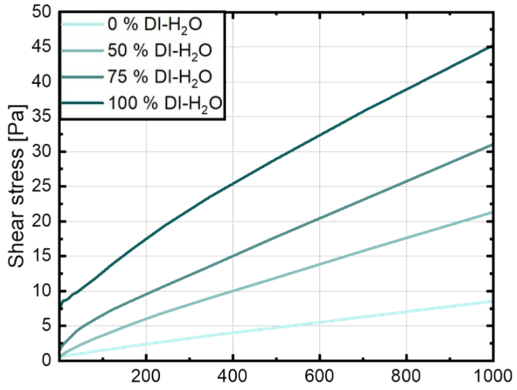

S3 Shear stress curves of inks with different water contents.

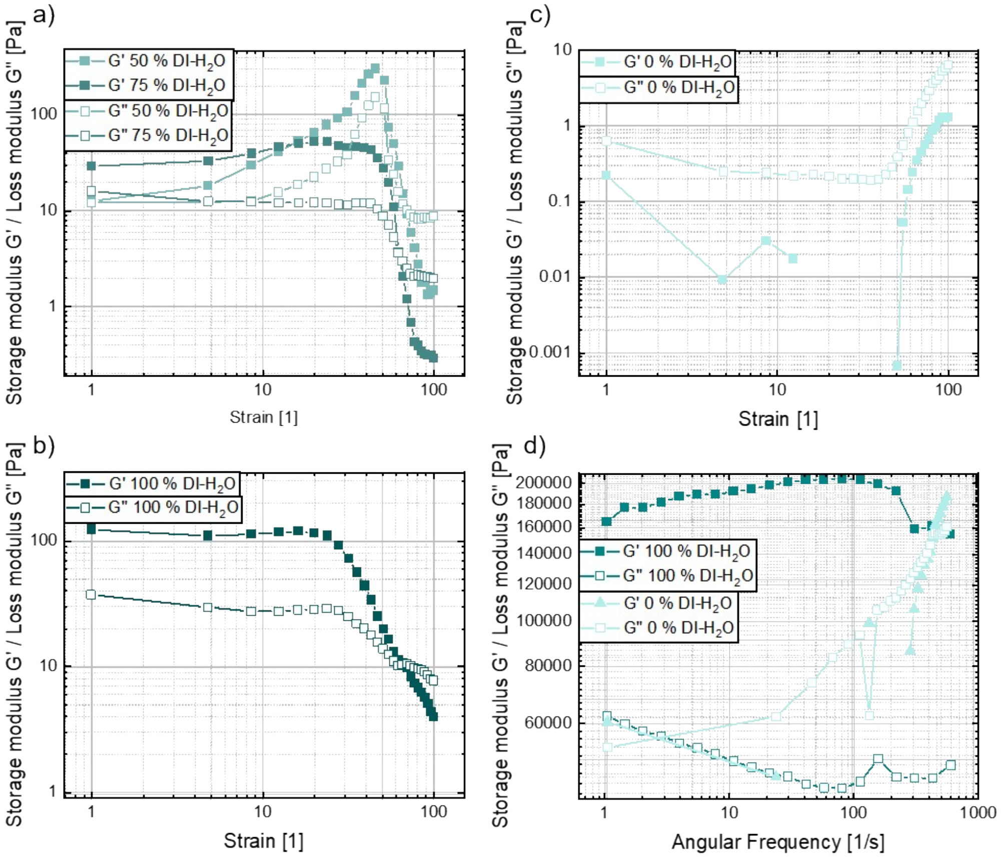

S4 Amplitude scans of inks with different water contents (a-c), frequency scan of only water and only IPA inks (d).

Morphological analysis of the catalyst layers

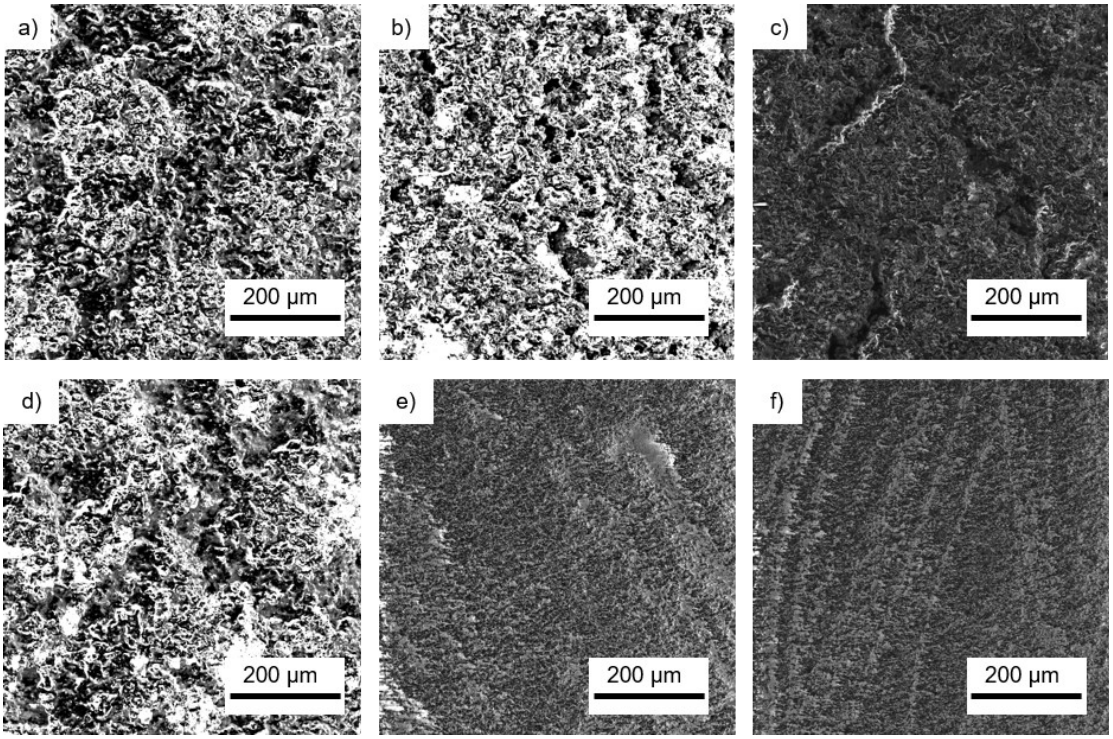

S5 SEM images of anode catalyst layers with inks mixed at 30 rpm for 4 hours (a), 24 hours (b), and 48 hours (c), (d-f) catalyst layers of inks mixed at 80 rpm for 4 hours (a), 24 hours (b), and 48 hours (c). Images taken using SEM Maia 3 with SE detector.

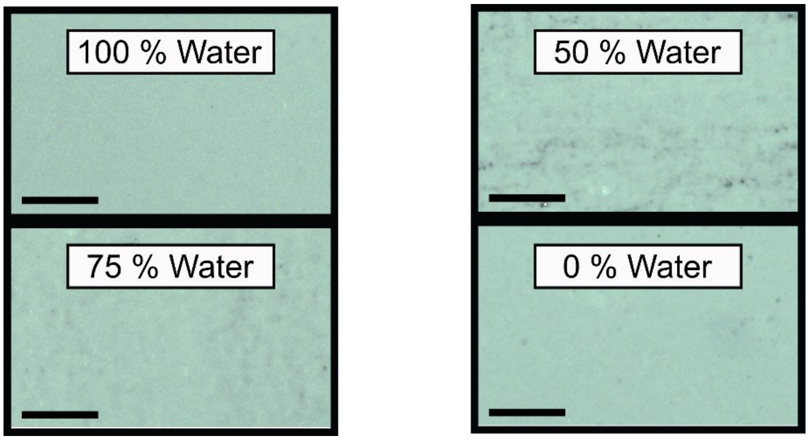

S8 Microscopic images of parts of the HCCMs fabricated using the inks with water content variation. . Images taken with a confocal microscope from WiTeC using a 5x, Zeiss EC "Epiplan" objective. Black bars represent a scale of 500 µm.

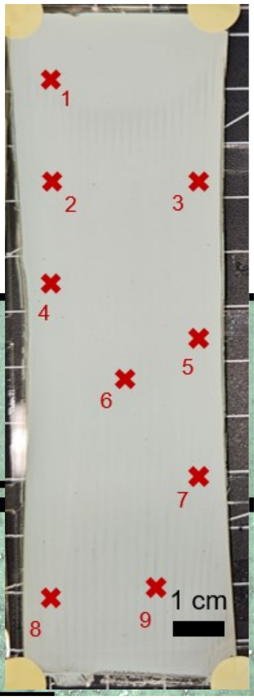

Point	Loading (mg_{Ni}/cm^2)
1	0.80
2	0.80
3	0.85
4	0.76
5	0.75
6	0.75
7	0.81
8	0.76
9	0.75
Mean	0.78
Std Dev	0.03

S7 A fully coated HCCM (75 % water ink), with an area of 48 cm2, red crosses approximately depict the XRF measuring points and their respective loading shown in the table. This HCCM is an example of a homogeneous catalyst layer with a standard deviation of 0.03. The points were chosen randomly and each point was measured separately with refocusing to minimize the measurement errors.

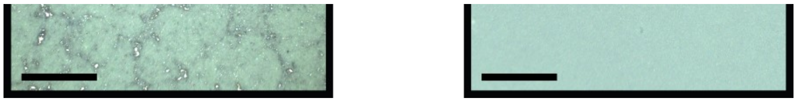

S6 Microscopic images of parts of the HCCMs fabricated using the inks with roller mixer parameter variation. Images taken with a confocal microscope from WiTeC using a 5x, Zeiss EC "Epiplan" objective. Black bars represent a scale of 500 µm. The images depict the agglomeration scale within the catalyst layers as well as the number and size of pores.
